# Automated Landslide Identification from Time-Series InSAR Using Improved Hot Spot Analysis

**DOI:** 10.3390/s26092771

**Published:** 2026-04-29

**Authors:** Xiaoxiao Yang, Jinmin Zhang, Wu Zhu, Quan Sun, Jing Li

**Affiliations:** 1 Aerial Photogrammetry and Remote Sensing Group Co., Ltd., Xi’an 710199, China; yang_quixote@163.com (X.Y.); 2020126026@chd.edu.cn (Q.S.); lijing07120508@163.com (J.L.); 2 School of Geological Engineering and Geomatics, Chang’an University, Xi’an 710054, China; zhuwu@chd.edu.cn

**Keywords:** automated identification, landslide, improved hot spot analysis, InSAR, Sentinel-1A, IPTA-SBAS

## Abstract

**Highlights:**

**What are the main findings?**

- An improved hot spot analysis (IHSA) method with multi-weight factor coupling is proposed for high-precision automatic landslide extraction using time-series InSAR data.
- The IHSA method significantly outperforms the traditional Getis-Ord Gi* model, achieving 90.20% precision and 92.00% recall, while eliminating over-segmentation and boundary defects in landslide extraction.

**What are the implications of the main findings?**

- This training-free method provides an efficient, reliable technical solution for large-scale early landslide identification in the complex geological environment.
- The multi-weight coupling framework has strong scalability, offering a methodological reference for automatic landslide identification in other geohazard-prone regions.

**Abstract:**

To address the key limitations of traditional automated landslide detection methods—namely their reliance on large training datasets, insufficient detection accuracy, and high false positive rates—this study proposes an InSAR-based automated landslide detection approach integrating multi-weight factor coupling, referred to as an Improved Hot Spot Analysis (IHSA) method. Built upon InSAR-derived surface deformation data, the proposed method optimizes the hotspot detection model through a spatial weighting matrix that incorporates multi-feature fusion. Morphological processing is further applied to refine landslide boundaries. Validation against manually interpreted ground truth data demonstrates that the proposed method achieves a precision of 90.20%, representing an improvement of 53.61 percentage points over the conventional hotspot analysis method, while maintaining a stable recall rate of 92.00%. The extracted landslide boundaries exhibit high consistency with manual interpretation results, effectively overcoming common issues in traditional approaches such as fragmented outputs and internal voids. This study provides an efficient, training-free solution for large-scale early identification of potential landslides, offering critical methodological support and data foundations for regional landslide detection and hazard mitigation.

## 1. Introduction

Landslides are among the most destructive and frequently occurring geological hazards worldwide, posing severe threats to the safety of mountain communities, critical infrastructure, and engineering facilities [1]. Therefore, high-accuracy and high-efficiency automated landslide detection is a fundamental prerequisite for regional landslide hazard assessment, monitoring and early warning, and emergency response [2]. With the rapid development of remote sensing technologies, Interferometric Synthetic Aperture Radar (InSAR) has become a key technique for regional landslide identification and surface deformation monitoring due to its unique advantages, including all-weather and day-night observation capability, wide spatial coverage, and sub-centimeter to millimeter-level deformation measurement accuracy [3,4,5,6,7].

In recent years, significant progress has been made in InSAR-based automatic landslide detection methods, which can generally be categorized into three major groups: threshold-based methods, machine learning methods, and spatial clustering methods. Threshold-based methods directly identify anomalous deformation areas by applying predefined deformation thresholds. Although computationally efficient and conceptually simple, they are highly sensitive to noise and show limited adaptability in complex terrains [8]. Machine learning methods represented by convolutional neural networks (e.g., the YOLOv7 deep learning model [9]) and random forests can automatically extract landslide information by learning multi-dimensional features and generally achieve high identification accuracy [10,11]. However, these methods typically rely on large-scale labeled datasets for model training and exhibit limited generalization ability across regions with distinct geological settings and geomorphic conditions, resulting in unstable detection performance in different study areas [12,13].

As a widely adopted technical approach for identifying deformation anomalies with spatial aggregation characteristics, spatial clustering methods have been extensively applied in automated landslide detection. However, traditional spatial hotspot analysis models still exhibit obvious limitations. On the one hand, the classical Getis-Ord Gi* hotspot analysis constructs spatial weights solely based on Euclidean distance, assigning equal weights to all deformation pixels and ignoring the differences in InSAR monitoring quality caused by temporal decorrelation [14,15]. On the other hand, conventional hotspot models fail to fully consider the spatial pattern of landslide deformation itself and the constraints imposed by terrain conditions. This deficiency often results in the misclassification of discrete noise pixels and anomalous scattering points as landslide areas, leading to substantial misidentifications [16].

To address the above limitations, this study focuses on non-instantaneous slow-moving landslides that can be reliably captured by time-series InSAR, including creep landslides, translational/rotational slides, and reactivated relict landslides. On this basis, we propose an improved hotspot analysis method (IHSA), which couples InSAR coherence coefficients with multi-dimensional landslide deformation characteristics to achieve automatic landslide identification. The proposed method breaks through the inherent bottlenecks of traditional hotspot analysis models and realizes high-precision automated extraction of potential landslides.

The method is validated in the main stream of the Yangtze River, a typical landslide-prone region with complex geological settings, steep topographic relief, and intensive human engineering activities [17,18]. The core conclusions are summarized as follows:

(1) Compared with the conventional Getis-Ord Gi* hotspot analysis, the proposed IHSA method significantly suppresses false positive interference, with the overall precision increasing from 36.59% to 90.20% (an improvement of 53.61 percentage points), while the recall remains stable at 92.00%, achieving an optimal balance between the completeness and accuracy of landslide detection; 

(2) The proposed method can produce continuous and smooth landslide boundaries, effectively solving the prevalent defects of jagged edges, fragmented patches and internal voids in the extraction results of traditional methods.

## 2. Materials

### 2.1. Study Area

**Figure 1:**
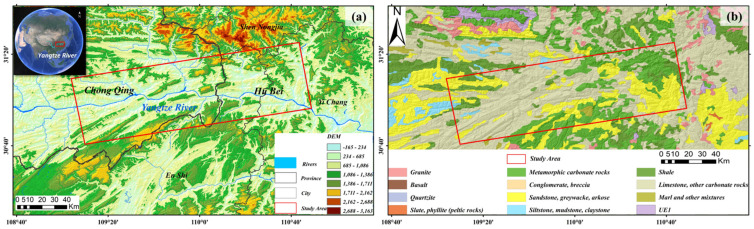
(**a**) Enlarged view of the target region; (**b**) Lithological Map of the Study Area.

The study area is located along the main stream of the Yangtze River at the boundary between Chongqing Municipality and Hubei Province, China, with geographical coordinates of approximately 109°00′–110°50′ E and 30°40′–31°20′ N. This region covers a key transitional section along the Yangtze River and represents one of the typical areas within the Yangtze River Economic Belt characterized by complex geological environments and frequent landslide hazards [19]. The regional landform is dominated by middle–low mountainous terrain. The western part represents the eastern extension of the Daba Mountains and Wushan Mountains, where the topography is steep and dissected by dense valleys [20,21]. Toward the east, the terrain gradually transitions to the marginal zone of the Jianghan Plain, where the topography becomes relatively gentle. The main stream of the Yangtze River flows across the entire region from west to east (Figure 1a). Long-term river incision has formed deeply incised canyon landscapes, and slopes on both sides of the river valley generally range from 15° to 45°, providing favorable geomorphological conditions for the development of landslides [22]. The study area is dominated by carbonate rocks (mainly limestone), with sandstone as the secondary lithology. Subjected to long-term regional tectonic activities and fluvial incision by the Yangtze River, the rock masses exhibit well-developed karst fissures, highly fragmented bedrock, and dense joint networks. The hard-soft interbedded lithological combination, coupled with locally distributed argillaceous weak interlayers, further deteriorates the overall stability of slopes (Figure 1b), thus forming a favorable geological prerequisite for landslide occurrence [23]. The region is characterized by a subtropical monsoon climate with an average annual precipitation of approximately 1000–1500 mm. Rainfall distribution is highly uneven and mainly concentrated in summer (May–September), often occurring as continuous heavy rainfall or short-duration intense storms. Rainfall infiltration significantly increases soil weight and reduces shear strength, making precipitation the most important natural triggering factor for landslides [24]. In addition, after the impoundment of the Three Gorges Reservoir, periodic fluctuations in reservoir water levels cause repeated wetting–drying cycles along the reservoir banks. Variations in pore water pressure and soil softening further aggravate slope instability [25]. Furthermore, human engineering activities such as unreasonable slope cutting and loading disturb the natural stress balance of slopes, thereby increasing the probability of landslide occurrence [26]. Overall, landslides in this region are predominantly distributed in a belt-like pattern along the main stream of the Yangtze River and its tributaries, with particularly high concentrations in deeply incised canyon sections. Their development and evolution are jointly controlled by multiple factors, including geomorphology, geological structure, rainfall, reservoir water-level fluctuations, and human engineering activities.

### 2.2. Data

Launched in 2014 by the European Space Agency (ESA), the Sentinel-1A satellite provides open-access C-band SAR data acquired via the Terrain Observation with Progressive Scans (TOPS) imaging mode [27]. With a 12-day revisit cycle and extensive long-term archived datasets, it is widely adopted for geohazard-related surface deformation monitoring, despite limitations including coarse spatial resolution, limited vegetation penetration, and non-programmable acquisition scheduling [28,29]. In this study, we used Sentinel-1A SAR data of Path 157 and Frame 92, covering the period from April 2015 to February 2025; the specific acquisition parameters are given in Table 1 and Table 2. All raw SAR images were subjected to standard preprocessing procedures including orbit refinement, resampling and coregistration, to support the subsequent data processing workflow.

## 3. Methods

### 3.1. IPTA-SBAS

Among the existing InSAR techniques capable of retrieving time-series surface deformation over large areas, Small Baseline Subset InSAR (SBAS-InSAR) [30] and Persistent Scatterer InSAR (PS-InSAR) [31] are the two most widely used approaches. The Small Baseline Subset (SBAS) technique, proposed by Berardino in 2002, is a differential interferometric SAR method designed to efficiently retrieve continuous surface deformation information. Its core principle is to divide all SAR images into multiple short-baseline subsets according to predefined temporal and spatial baseline thresholds. Interferometric processing is then performed for each subset to obtain deformation measurements [32,33]. A key advantage of the SBAS technique lies in its relatively low requirement for SAR data volume. Meanwhile, strict constraints on temporal and spatial baselines effectively mitigate the impacts of decorrelation and topographic effects on deformation retrieval.

The Interferometric Point Target Analysis (IPTA) technique was developed based on the PS-InSAR framework. Werner designed the IPTA processing module in the GAMMA software (version 2023), which allows flexible configuration of either single-master or multi-master processing modes. Through regression analysis and multiple iterative procedures, various error sources can be progressively reduced to obtain high-precision spatiotemporal surface deformation information [34]. The basic principle involves selecting targets with stable backscattering characteristics and applying one-dimensional or two-dimensional regression analysis to estimate linear deformation, DEM error, and residual components for each target point [35,36]. After correcting DEM errors, spatiotemporal filtering is applied to separate atmospheric delay and nonlinear deformation components from the residuals, thereby retrieving the time-series deformation characteristics of each target point. Through multiple iterations, the influence of decorrelation noise, atmospheric delay, and DEM errors is gradually suppressed [37,38]. Compared with the SBAS approach, the limitation of IPTA is that it mainly focuses on high-coherence stable scatterers for interferometric processing and time-series inversion, making it difficult to obtain the complete spatial extent and morphological characteristics of geological hazards. Therefore, combining SBAS and IPTA enables complementary advantages, allowing more accurate and spatially continuous surface deformation results to be obtained.

**Figure 2:**
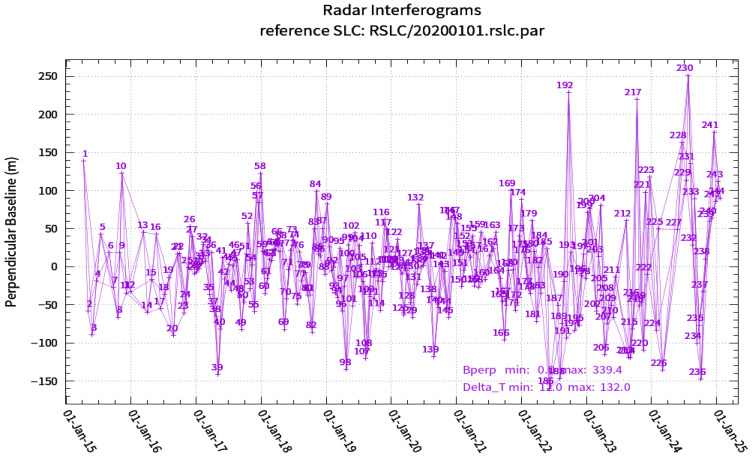
Diagram of the Spatiotemporal Baselines.

**Figure 3:**
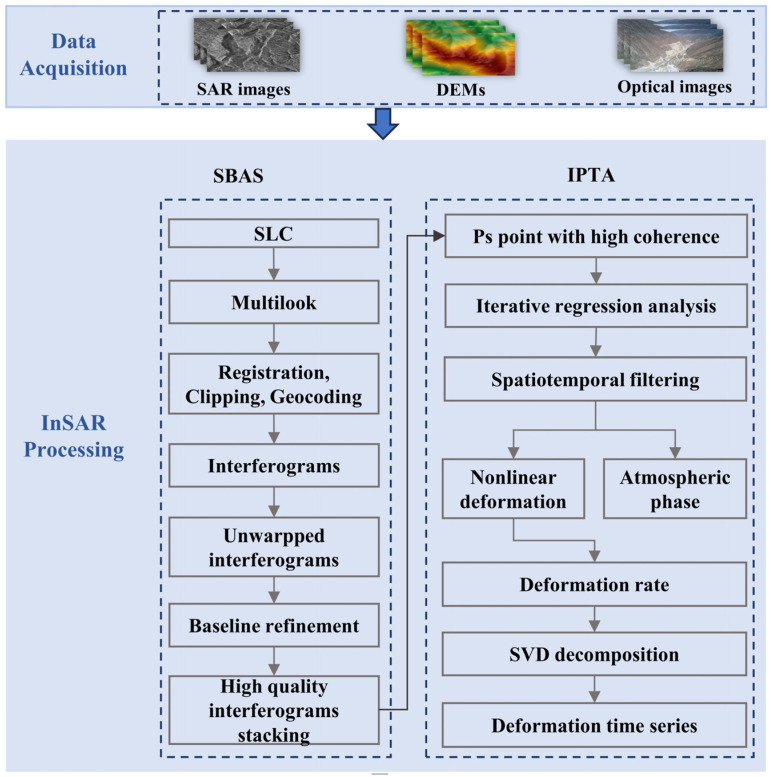
Flowchart of the IPTA-SBAS Time-Series InSAR Technique.

In this study, a time-series InSAR technique integrating the Small Baseline Subset (SBAS) and Interferometric Point Target Analysis (IPTA) was adopted for data processing. This combined approach retains the flexible interferometric pair combination capability of SBAS, while leveraging the iterative processing characteristics of IPTA to derive continuous, high-precision deformation results. For data support, the 30 m-resolution Shuttle Radar Topography Mission (SRTM) Digital Elevation Model (DEM) was used to remove topographic phase contributions, and 30 m-resolution optical imagery from Google Earth was employed for visual interpretation. For interferometric processing, the spatial and temporal baseline thresholds were set to 250 m and 36 days, respectively (Figure 2), generating a total of 726 valid interferometric pairs, among which 527 high-quality pairs were selected for subsequent inversion. Multi-looking interferometry, spatiotemporal baseline threshold constraint, and phase filtering were applied to greatly mitigate the impacts of atmospheric errors and decorrelation noise on the inversion results. Meanwhile, a coherence threshold of 0.3 was set to mask low-quality regions. Finally, we inverted the Line-of-Sight (LOS) surface deformation rate and the nearly decadal time-series deformation sequence of the study area from April 2015 to January 2025. The overall data processing workflow is illustrated in Figure 3.

### 3.2. Automatic Extraction of Landslide Deformation

Landslide deformation derived from time-series InSAR is controlled by the slip surface and typically exhibits continuous areal deformation, resulting in strong spatial correlation among neighboring monitoring points [39]. In contrast, atmospheric delay, decorrelation noise, and non-landslide deformation generally show discrete and randomly distributed patterns without stable spatial correlation [40,41]. The traditional Getis–Ord Gi* statistic defines spatial association solely based on distance, without considering the reliability of InSAR observations or the physical characteristics of landslide deformation, which may lead to high false-positive detections [42,43]. To address this limitation, this study constructs a three-factor coupled spatial weight matrix by integrating spatial proximity, observation quality, and deformation-field integrity. This adaptive weighting scheme assigns higher weights to pixel pairs that are spatially close, have reliable deformation measurements, and belong to the same landslide body, thereby improving hotspot detection accuracy.

#### 3.2.1. Preprocessing

Landslide occurrence exhibits significant topographic dependence, with its spatial distribution strongly constrained by slope conditions. Statistics from the historical landslide inventory in the study area indicate that over 90% of landslides are developed within the slope range of 10° to 45°, whereas gentle slopes below 10° have a landslide occurrence probability of less than 1% and are thus defined as non-landslide-prone areas. River water bodies lack the geological conditions for landslide development, and SAR signals suffer from severe decorrelation over water surfaces, rendering the corresponding deformation results geologically meaningless. In addition, the coherence coefficient is the core quantitative metric for assessing the reliability of time-series InSAR deformation results; deformation results in low-coherence regions (*γ* < 0.3) are highly vulnerable to phase noise contamination and have no valid utility [2].

To mitigate the risk of false positive misdetections from stable and noisy regions at the source, we uniformly masked out invalid pixels with slopes < 10°, average coherence *γ* < 0.3, and all river water body areas in this preprocessing step. Only valid monitoring points that meet the geological conditions for landslide development and have reliable deformation results were retained for the subsequent spatial statistical analysis.

#### 3.2.2. Construction of the Coupled Spatial Weight Matrix

(1) Distance Weight $W_{s}$

Distance weight defines the basic spatial association rule, where closer neighboring points receive higher weights within a defined spatial neighborhood:

(1) $$W_{s}=\left\{\begin{array}{ll} \frac{1}{d_{ij}^{2}}, & d_{ij}\leq d_{0}\cup j\neq i\\ 0, & d_{ij}>d_{0}\cap j=i \end{array}\right.$$

where $d_{ij}$ is the Euclidean distance between target point i and neighboring point *j*, and $d_{0}$ is the optimal analysis distance determined using Global Moran’s I.

(2) Coherence-Based Quality Weight $w_{\gamma }$

Based on the classic InSAR theory [2] and the general specifications of Sentinel-1 InSAR landslide studies, the deformation results have no valid application value when the coherence coefficient *γ* < 0.3, and *γ* = 0.5 is taken as the demarcation threshold for high-confidence coherence. Combined with the topographic and vegetation characteristics of the deeply incised Yangtze River valley in the study area, this paper constructs a differentiated coherence quality weight $W_{\gamma }$ for each monitoring point: a weight of 0 is assigned to eliminate noise when *γ* < 0.3, linear weighting is adopted for points with 0.3 ≤ *γ* ≤ 0.5, and a full weight of 1 is assigned to high-confidence deformation points when *γ* > 0.5. This design can amplify high-confidence deformation signals, suppress noise interference, and fundamentally reduce the misjudgment of false hotspots. The weighting rules are as follows:

(2) $$W_{\gamma }=\left\{\begin{array}{l} 0,\;\gamma_{i}<0.3\\ \gamma_{i},\;0.3\leq \gamma_{i}\leq 0.5\;\\ 1,\;\gamma_{i}>0.5\; \end{array}\right.$$

(3) Deformation Integrity Weight $W_{n}$

During the creeping stage, a landslide behaves as a continuous deforming body constrained by the basal slip surface. Therefore, deformation rates within the same landslide body exhibit minimal relative deviation and consistent deformation trends.

The relative deviation in deformation rate is defined as:

(3) $$\delta_{ij}=\frac{\left|x_{i}-x_{j}\right|}{|x_{i}|+|x_{j}|+\varepsilon }$$

where $x_{i}$ and $x_{j}$ represent the annual deformation rates of points *i* and *j*, respectively, and ${\varepsilon =10}^{-5}$ mm/yr is a small constant to avoid division by zero.

The deviation is mapped to a weight using a negative exponential function:

(4) $$W_{n}=exp\left(-\delta_{ij}\right)$$

(4) Weight Coupling

The three weighting factors are integrated using multiplicative coupling:

(5) $$W_{ij}=W_{s}\times W_{\gamma }\times W_{n}$$

(5) Row Standardization

To ensure comparability among different monitoring points and satisfy the requirements of the Gi* statistic, the initial weight matrix is row-standardized:

(6) $$w_{ij}\left(d_{0}\right)=\frac{w_{ij}^{\mathrm{initial}}\left(d_{0}\right)}{\sum_{j=1}^{n}\;\;w_{ij}^{\mathrm{initial}}\left(d_{0}\right)}$$

After normalization, the sum of weights in each row equals 1, ensuring consistent statistical interpretation.

#### 3.2.3. Getis–Ord Gi* Statistic and Hotspot Identification

(1) Determination of the Optimal Analysis Distance

The optimal spatial scale for clustering analysis is determined using incremental spatial autocorrelation based on the Global Moran’s I statistic [5]:

(7) $$I=\frac{n}{S_{0}}\frac{\sum_{i=1}^{n}\;\sum_{j=1}^{n}\;w_{ij}\left(x_{i}-\bar{x}\right)\left(x_{j}-\bar{x}\right)}{\sum_{j=1}^{n}\;{\left(x_{j}-\bar{x}\right)}^{2}}$$

where *n* is the total number of features in spatial clustering, *x* is the mean value of all features, $w_{ij}$ is the spatial weight between features, and $S_{0}$ is the sum of all spatial weights.

(2) Improved Gi* Statistic

After determining the optimal distance threshold, the Gi* statistic is applied to detect spatial clustering of deformation values:

(8) $$G_{i}^{*}(d)=\frac{\sum_{j=1}^{n}\;\;w_{ij}(d)x_{j}}{\sum_{j=1}^{n}\;\;x_{j}}$$

At the 99% confidence level, pixels with z > 2.58 and *p* < 0.01 are identified as significant hotspots (landslide deformation clusters), while z < −2.58 indicates cold spots corresponding to stable areas.

#### 3.2.4. Landslide Boundary Extraction and Optimization

**Figure 4:**
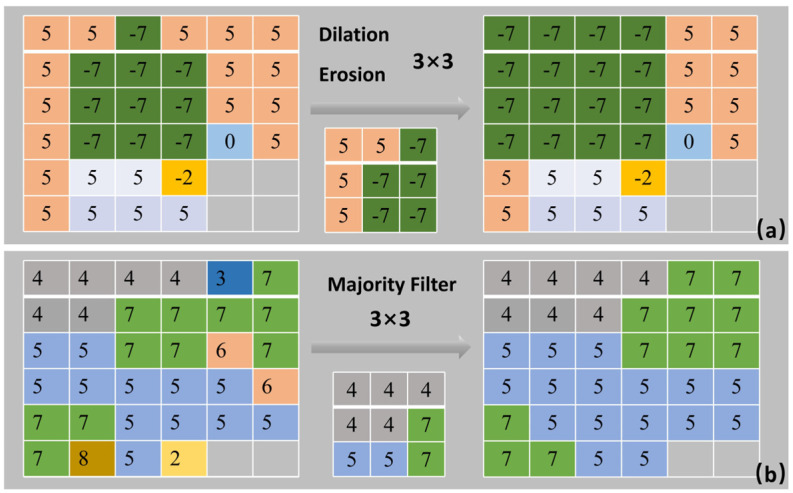
Schematic Diagram of Boundary Optimization. (**a**) Schematic diagram of morphological operations; (**b**) Schematic diagram of majority filtering.

Based on the landslide deformation hotspots derived from the improved Gi* statistics, we achieved accurate extraction and optimization of landslide boundaries via three sequential core steps:

1. Initial Boundary Extraction: We first performed binarization segmentation of the hotspot raster under the 99% confidence level significance threshold, then applied 8-neighborhood spatial connectivity analysis to aggregate discrete hotspots into continuous planar landslide candidate patches. To further reduce false-positive misjudgments caused by low signal-to-noise ratio micro-patches, we eliminated patches with an area smaller than 0.01 km^2^ according to the spatial resolution of Sentinel-1A data and the landslide development characteristics of the study area, thus obtaining the initial landslide boundaries.
2. Morphological Structure Optimization (Figure 4a): We adopted mathematical morphological opening and closing operations with a 3 × 3 structural element [44] to merge scattered boundary pixels and remove false-positive fragmented patches. An area-based descending priority rule was synchronously introduced to prioritize the restoration of the complete spatial morphology of large-area landslides.
3. Boundary Smoothing Processing (Figure 4b): A 4-neighborhood mode filtering algorithm [45] was applied to implement pixel-level smoothing of the landslide boundaries, which repaired fine boundary serrations while maximally preserving the original structural features of the landslide boundaries.

**Figure 5:**
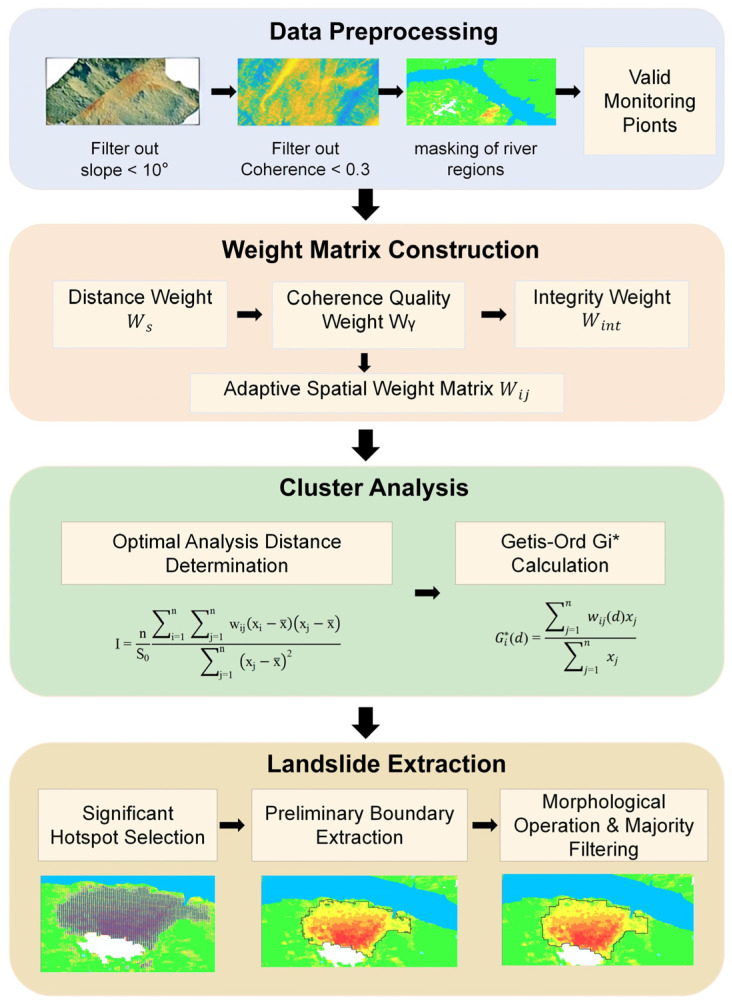
Overall Technical Flowchart of the Proposed IHSA Method.

Through the above workflow, we finally obtained the vector landslide boundaries and landslide inventory with continuous smooth boundaries and complete spatial structure. The proposed workflow effectively overcomes the shortcomings of fragmented results and over-segmentation in traditional Gi* methods and significantly improves the identification accuracy of landslide boundaries. The overall workflow of automatic landslide extraction is shown in Figure 5.

## 4. Results and Discussion

### 4.1. Results of Deformation Monitoring and Landslide Identification

**Figure 6:**
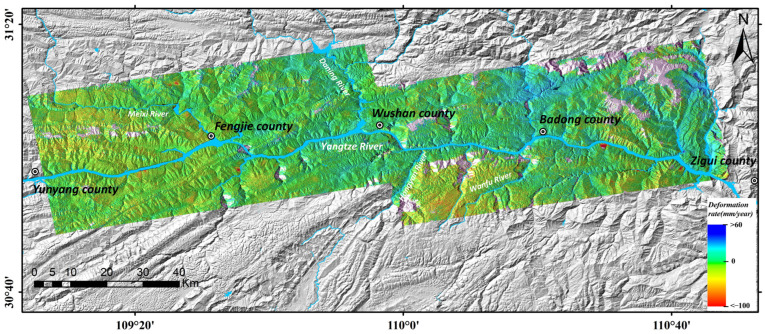
Map of Annual Mean LOS Surface Deformation Rate.

The spatial distribution of the annual mean LOS surface deformation rate is shown in Figure 6. In this study, areas with an annual mean deformation rate within ±10 mm/yr were defined as stable regions (displayed in green in the figure). Positive deformation values (blue) indicate surface movement towards the satellite, while negative values (red) represent movement away from the satellite (dominated by ground subsidence). Regarding the overall spatial distribution, effective monitoring results were obtained for most areas except for a few ridges with high altitude and dense vegetation cover, with the deformation rates ranging from −71 mm/yr to +44 mm/yr. Notable atmospheric phase delay errors are observed north of Badong County and along both banks of the Wanfu River, which is presumably attributed to the uneven spatiotemporal distribution of water vapor driven by the deeply incised river valleys and subtropical monsoon climate in this region. Overall, the central part of the study area is relatively stable, while more deformed areas are distributed in the eastern and western sections. Large-scale concentrated surface deformation zones mostly occur in a banded pattern along the bank slopes of major water systems, including the main stream of the Yangtze River, Daning River, and Meixi River.

**Figure 7:**
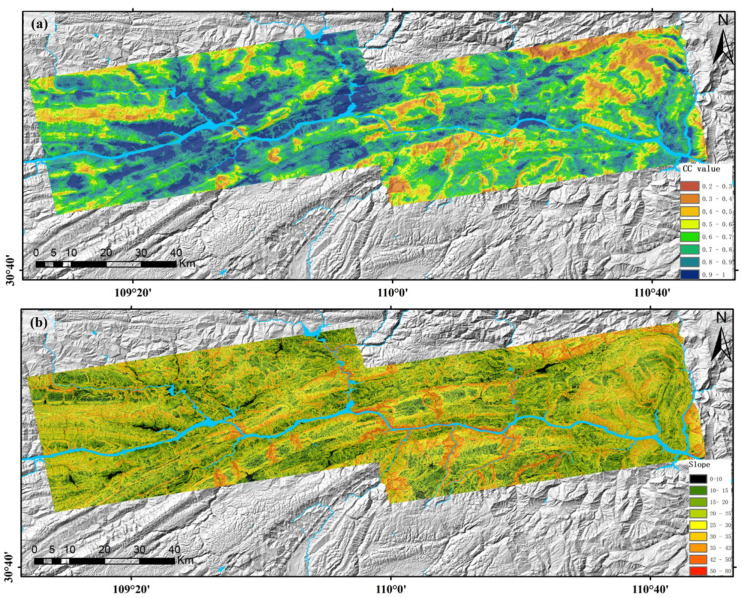
Maps of Coherence Coefficient and Slope. (**a**) Coherence coefficient map; (**b**) Slope map.

**Figure 8:**
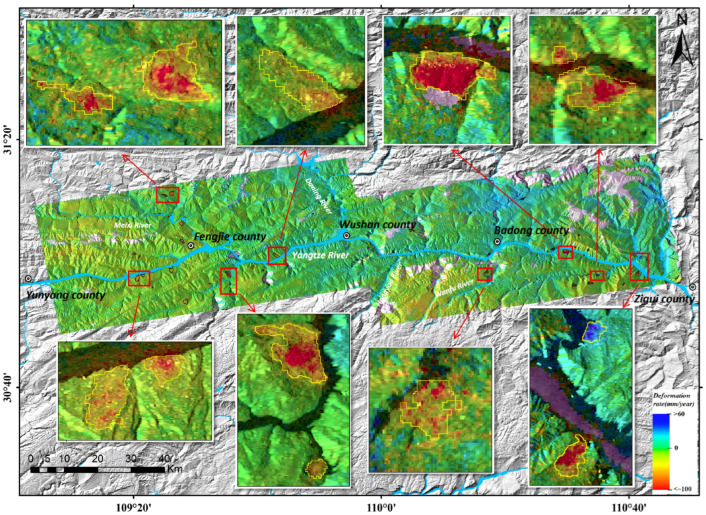
Results of Automatic Landslide Extraction.

**Figure 9:**
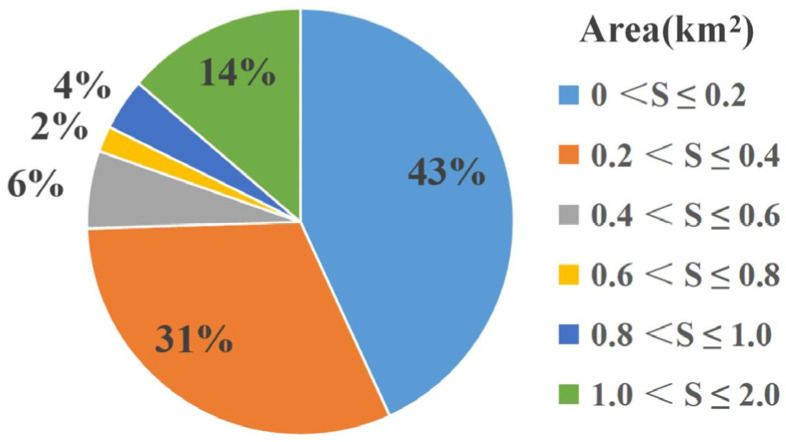
Landslide area proportion chart.

Subsequently, preprocessing was performed on the obtained annual mean deformation rate results, including masking of areas with coherence < 0.3, slope < 10°, and river water bodies (the coherence map and slope map of the study area are shown in Figure 7). A coupled weight matrix was constructed by integrating the coherence constraint, landslide mass integrity constraint, and spatial proximity constraint, and the Improved Hot Spot Analysis (IHSA) method was adopted to realize automatic extraction of landslide areas. After post-processing with morphological operations and majority filtering (both with a 3 × 3 kernel), a total of 51 effective potential landslide sites were identified, with the extraction results shown in Figure 8. The enlarged view of typical landslide extraction results preliminarily reveals that the automatically delineated landslide boundaries are highly consistent with the actual monitoring results. As shown in Figure 9, the landslides extracted by the proposed method are dominated by small and medium-sized landslides. This size distribution pattern is consistent with the universal frequency-area distribution law of landslides revealed by existing regional-scale studies [46]. Specifically, landslides with an area of ≤0.4 km^2^ collectively account for 74% of the total landslide inventory, while large-scale landslides with an area > 1.0 km^2^ account for 14%. This distribution is in full agreement with the landslide development characteristics of the study area and the design expectation of the proposed method. For this calculation, a total of 8.37 million deformation vector points were involved. The computation was performed on a platform equipped with a 12th Generation Intel Core i7 CPU and 64 GB RAM, with a total processing time of approximately 0.5 h, demonstrating high computational efficiency.

### 4.2. Comparative Analysis of Landslide Identification Counts

To verify the accuracy of the improved hot spot analysis (IHSA) method for automatic landslide identification and clarify its optimization performance over traditional methods, this study conducted a comparative analysis using manual delineation results as the ground truth. We performed a systematic comparison among the original traditional Getis-Ord Gi* method, the proposed improved IHSA method, and the manual delineation results. The reliability and superiority of the automatic identification method were comprehensively evaluated from three dimensions: quantitative accuracy, spatial matching degree, and details of typical cases, to provide data support for the engineering application of the method. During the comparison, a 99% confidence level (Z > 2.58, *p* < 0.01) was uniformly adopted as the identification criterion for landslides to ensure consistent comparison conditions between the traditional and improved methods, while micro landslides with an area less than 0.01 km^2^ were excluded.

**Figure 10:**
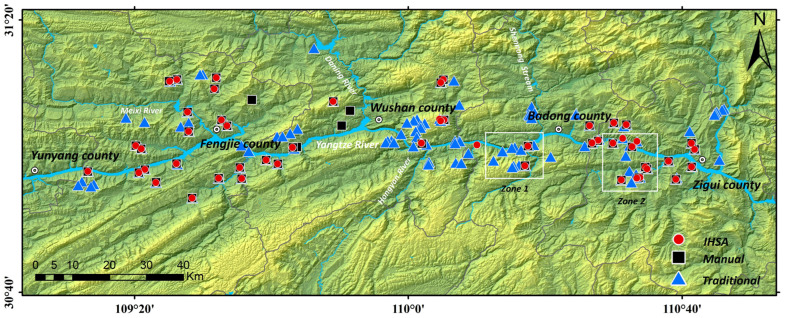
Comparison of Landslide Identification Results from Three Methods.

**Figure 11:**
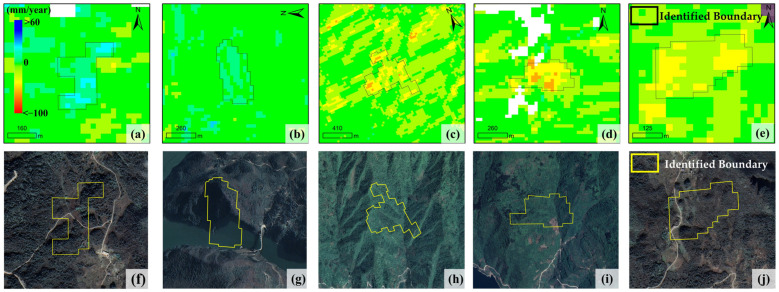
Falsely identified landslides: InSAR-derived deformation rate maps (**a**–**e**) and their corresponding optical images (**f**–**j**) with identified boundaries.

**Figure 12:**
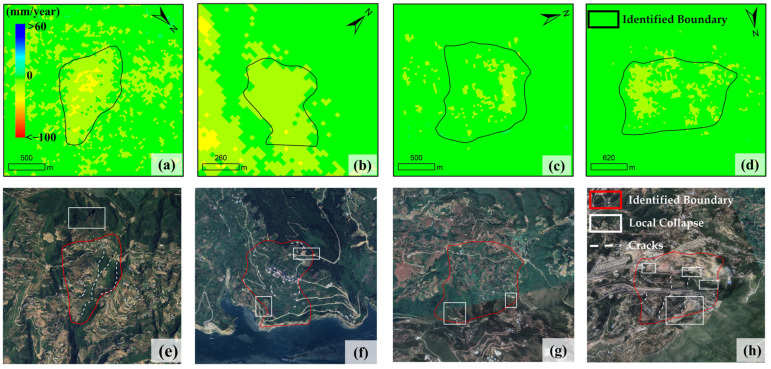
Missed landslides: InSAR deformation rate maps (**a**–**d**) and corresponding optical images (**e**–**h**) with manual interpretation.

A total of 50 landslides were extracted in the study area through manual interpretation and delineation by professional technicians. The comparison shows that the improved IHSA method automatically identified 51 landslides in total, including 5 false positives and 4 false negatives (omissions); in contrast, the unmodified traditional hot spot analysis method extracted 123 landslides, including 77 false positives and 5 false negatives (Figure 10). As shown in Figure 11, although the 5 falsely identified areas by the improved method exhibit certain spatial clustering characteristics in the deformation field, no obvious landslide deformation traces are found in the optical images. It is inferred that such false identifications are mainly caused by phase unwrapping errors and residual orbital errors during InSAR data processing. For the 4 missed landslides (Figure 12), although their deformation fields are relatively concentrated, the hot spot anomaly values are not prominent with no obvious high-value areas, which makes it difficult to meet the identification criteria and ultimately leads to missed detection. Manual interpretation confirms that these 4 landslides all have clear deformation features on optical images: slope cracks are marked with white dashed lines, and signs of local slope collapse and slumping are marked with white boxes.

**Figure 13:**
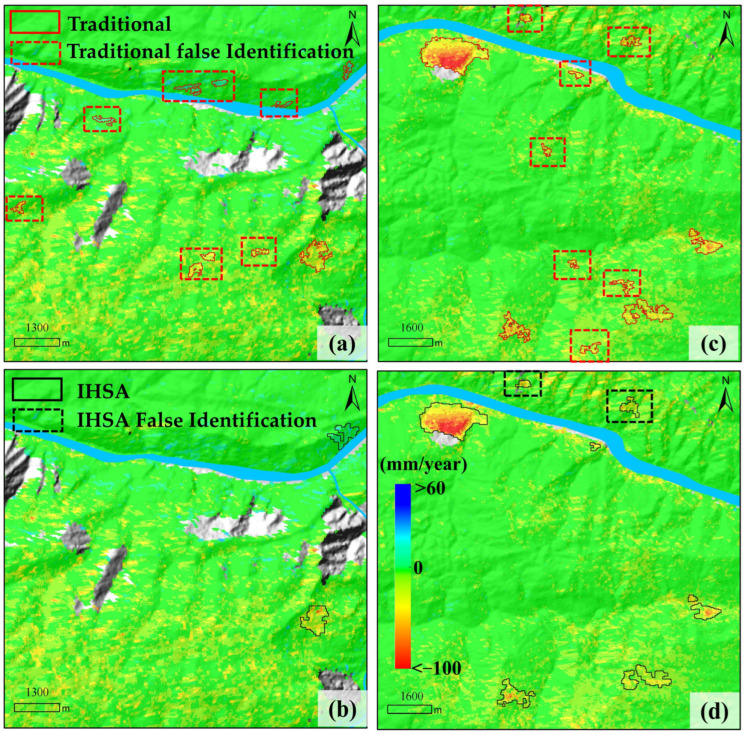
Detailed Comparison Between the Original Traditional Method (**a**,**b**) and the Improved IHSA Method (**c**,**d**).

The significant difference in the number of identifications between the traditional and improved methods is mainly reflected in the ability to suppress false positives. For this reason, two typical areas with concentrated false identifications (Area 1 and Area 2 in Figure 10) were selected for comparative analysis. As shown in Figure 13, the false identification areas of the traditional hot spot analysis are mostly distributed in regions with high vegetation coverage, poor coherence, and high susceptibility to unwrapping errors. Among them, black boxes represent the false positives of the traditional method, and red boxes represent those of the improved method. Overall, by constructing the coupled weight matrix, the improved IHSA method effectively filters out spurious deformation noise caused by decorrelation and unwrapping errors. While maintaining a favorable control level of the missed detection rate, it significantly reduces the number of false identifications, and its identification accuracy and reliability are obviously superior to the traditional method.

To further quantify the optimization effect of the proposed method, three quantitative accuracy metrics were adopted to evaluate the performance of the two automatic landslide identification methods from three dimensions: identification accuracy, identification integrity, and comprehensive performance. The definition, calculation logic, and formula of each metric are specified as follows:

Precision is used to measure the proportion of real landslides in the automatic identification results, which reflects the method’s ability to suppress false landslide detection. A higher value indicates fewer false identifications and stronger reliability of the results. The calculation formula is:

(9) $$Precision=\frac{TP}{TP+FP}$$

where TP (True Positive) is the number of correctly identified landslides, and FP (False Positive) is the number of falsely identified landslides.

Recall is used to measure the proportion of successfully identified landslides among the ground truth landslides, which reflects the method’s ability to avoid missed landslide detection. A higher value indicates fewer missed identifications and stronger integrity of the results. The calculation formula is:

(10) $$Recall=\frac{TP}{TP+FN}$$

where FN (False Negative) is the number of missed landslides.

F1-Score is the harmonic mean of Precision and Recall, which comprehensively characterizes the overall identification performance of the method. It avoids the one-sidedness of single-metric evaluation and resolves the inherent trade-off between Precision and Recall. The value ranges from 0 to 1, and a value closer to 1 indicates a better comprehensive identification performance. The calculation formula is:

(11) $$F1=2\times \frac{Precision\times Recall}{Precision+Recall}$$

**Figure 14:**
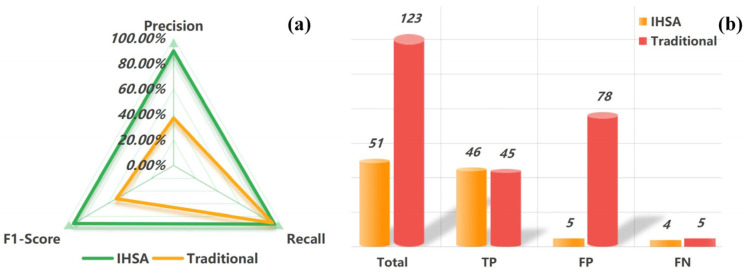
Performance comparison of the two methods. (**a**) Precision-recall-F1 radar chart; (**b**) Bar chart of TP, FP, FN and total landslides.

The quantitative results show that the improved IHSA method achieves a qualitative leap in comprehensive identification performance compared with the traditional method (Table 3 and Figure 14). For the core metrics, the Precision of the improved method reaches 90.20%, an increase of 53.61 percentage points compared with 36.59% of the traditional method. The Recall maintains a high level of 92.00%, meaning the method greatly improves the reliability of identification while ensuring the integrity of landslide detection. The contrast of the F1-Score (representing comprehensive performance) is even more prominent: the F1-Score of the improved IHSA method reaches 91.09%, while that of the traditional method is only 52.17%, with an improvement of 38.92 percentage points. This difference is mainly attributed to the large number of false identifications from the traditional method. In contrast, the improved method limits the number of false identifications to 5 through the coherence constraint and landslide mass integrity constraint, thus achieving a substantial enhancement in Precision.

### 4.3. Comparison of Identified Landslide Boundaries

**Figure 15:**
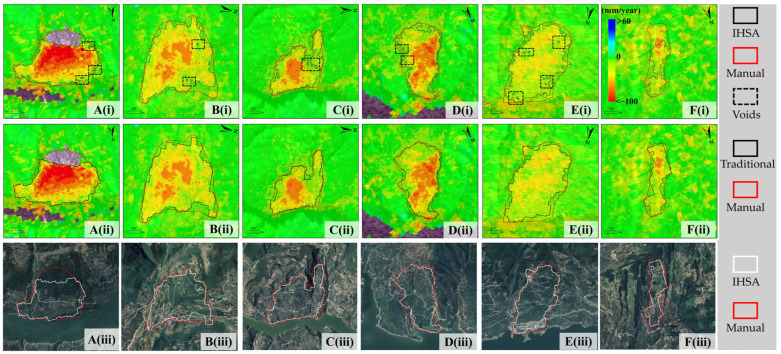
Comparison of landslide identification results from different methods for typical cases. (**A**–**F**) Six typical landslide cases. (i) IHSA identification results (black solid lines); (ii) Traditional method identification results (black solid lines); (iii) Optical images with boundaries from IHSA (white lines) and manual interpretation (red lines).

To further verify the accuracy of the IHSA method in landslide boundary extraction, 6 typical landslides were selected in this study to conduct a quantitative and qualitative comparison between the results of the IHSA method, the traditional method, and the optical image interpretation results (Figure 15).

In terms of boundary identification performance, the traditional method has two obvious limitations. First, it suffers from insufficient spatial integrity: it tends to fragment the same continuous landslide mass into multiple scattered and broken small patches, making it difficult to fully capture the overall extent of the landslide. As shown in Figure 15(Ei), the traditional method not only fails to effectively identify the upper area of the landslide but also incorrectly divides a single landslide into two parts, which is obviously inconsistent with the actual geological conditions. By introducing the landslide mass integrity constraint, the improved IHSA method effectively overcomes the over-segmentation problem and can completely and continuously identify the entire deformation area of the landslide (Figure 15(Eii)). Second, the extracted results have poor boundary smoothness and internal continuity. The boundaries extracted by the traditional method have many burrs, and a large number of fine internal voids are prone to appearing inside the extracted landslides. This phenomenon exists in typical slopes a, b, c, d, and f (marked by black boxes in the figure), with a maximum of 9 voids within a single landslide, which seriously affects the boundary accuracy and morphological rationality.

The comparison with the ground truth boundaries from manual delineation shows that the extraction results of the IHSA method are in high agreement with the manual interpretation results in terms of spatial distribution. It can delineate the deformation range of landslides more finely and completely, with smooth and continuous boundaries and intact internal structure, which is significantly superior to the traditional extraction method.

### 4.4. Analysis of a Typical Landslide

**Figure 16:**
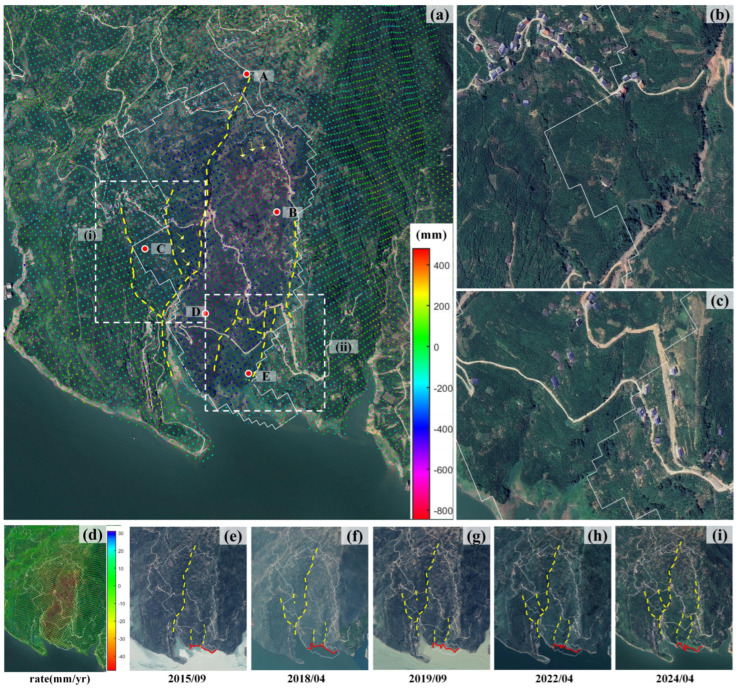
Detailed Illustration of the Typical Landslide. (**a**) Cumulative deformation map overlaid on the optical image; (**b**,**c**) enlarged views of the marked areas (i) and (ii) in (**a**), respectively; (**d**) annual mean deformation rate map; (**e**–**i**) Time-series optical remote sensing images of the landslide from 2015 to 2024.

To verify the accuracy and reliability of the proposed automatic landslide extraction method, a typical landslide in Hejialing Village, Zigui County, Hubei Province (along the main stream of the Yangtze River, center coordinates: 110.69° E, 30.974° N) was selected for validation. Figure 16a shows the nearly decadal cumulative surface deformation of the landslide from April 2015 to February 2025, and Figure 16d presents the corresponding annual mean surface deformation rate. The maximum cumulative deformation reaches ~850 mm in the middle of the right slope mass; the deformation is weakest on the left slope, followed by the landslide front edge. Obvious surface deformation signs are observed in optical images: a through-going fracture splits the surface rock-soil mass (Figure 16a), accompanied by multiple vertical secondary fractures (marked by yellow dashed lines), small collapses, and dense tension crack groups at the landslide rear edge. Enlarged details of the two highly deformed areas (i) and (ii) are shown in Figure 16b,c. Time-series optical images of the landslide from 2015 to 2024 (Figure 16e–i) show that the slope fractures (yellow dashed lines) develop significantly and increase in number over time. The actual outline of the landslide front edge is marked with a solid red line. The automatically extracted landslide boundary is highly consistent with the actual front outline, and the captured significant downslope creep of the front edge matches the deformation characteristics well, which fully validates the extraction accuracy and applicability of the proposed method.

To reveal the temporal deformation evolution of this landslide, 5 typical monitoring points (A–E) were deployed across the rear edge, middle part, and front edge of the landslide (Figure 16) to analyze the temporal deformation characteristics and driving mechanisms. The results show that all 5 monitoring points present an overall trend of continuous cumulative subsidence, with significant spatial heterogeneity in deformation features. Specifically, monitoring point E at the landslide front edge showed a notable phased uplift in July 2021, and the adjacent front-edge point D had a slight uplift response in the same period. In contrast, points A, B, and C at the middle and rear edges exhibited a synchronous accelerated subsidence trend. This deformation pattern is fully consistent with the typical evolution law of thrust-type landslides: the rock-soil mass at the middle and rear edges creeps and pushes downslope under gravity, compressing the front-edge mass to generate uplift deformation.

**Figure 17:**
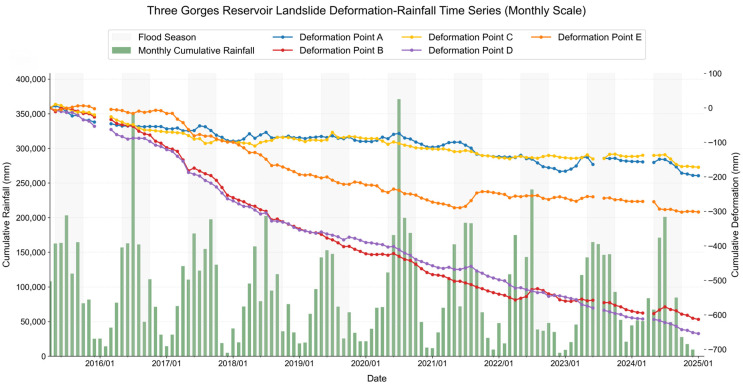
Time-Series Deformation Curves of Typical Monitoring Points. The location of the monitoring points in the figure is shown in Figure 15.

**Figure 18:**
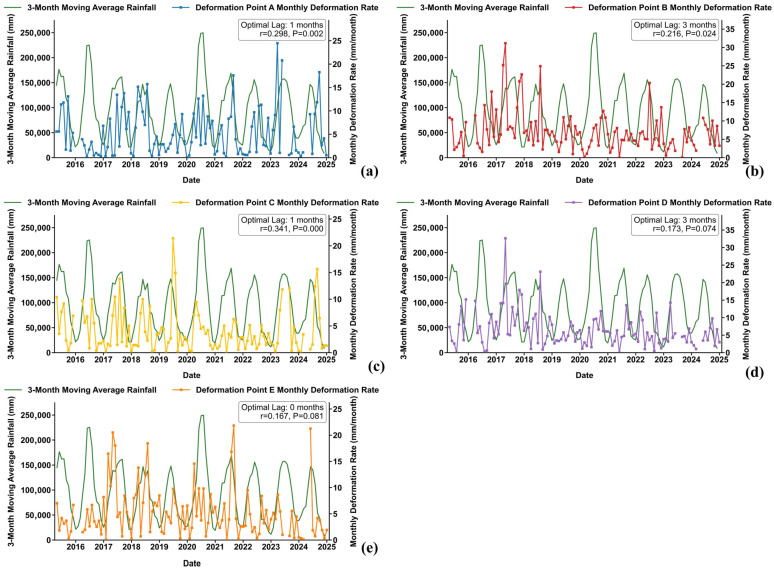
Correlation analysis between landslide deformation and rainfall. Panels (**a**–**e**) correspond to Deformation Points A, B, C, D, and E, respectively.

Furthermore, lag correlation and Pearson correlation coefficient analysis were performed, combined with the 3-month moving average rainfall of the study area [47] (Figure 17 and Figure 18). The deformation of points A, B, and C at the middle and rear edges is significantly correlated with rainfall, with a clear accelerated subsidence response after heavy rainfall events. Among them, points A and C show an obvious deformation response within 0–1 month after rainfall, confirming that rainfall is the core driving factor of deformation in this area. In comparison, the Pearson correlation coefficients between deformation and rainfall for front-edge points D and E are only 0.173 and 0.167, respectively, with both significance *p*-values > 0.05. This indicates that rainfall has no significant direct linear driving effect on the front-edge deformation, which is presumably mainly controlled by the overall thrust of the slope mass or other factors.

## 5. Conclusions

Aiming at the core limitations of insufficient identification accuracy and high false positive rate in traditional automatic extraction methods for unstable slopes, this study takes landslides along the main stream of the Yangtze River as the research object, and proposes an improved hot spot analysis method (IHSA) based on multi-weight factor coupling using time-series InSAR surface deformation data, to achieve high-precision automatic landslide extraction. The reliability of the method was systematically verified through comparative analysis with manual interpretation, ground truth and traditional methods, combined with time-series analysis of typical landslides. The main conclusions are as follows:

- A three-factor coupled spatial weight matrix was constructed, which effectively overcomes the limitation of the traditional Getis-Ord Gi* model that defines spatial association only by distance. This matrix couples three key factors: InSAR coherence, spatial integrity of the deformation field, and spatial aggregation characteristics of deformation anomalies, achieving the core goal of “assigning high weights to pixel pairs that are spatially adjacent, with high data quality, and belonging to the same landslide mass”. It enhances the real landslide deformation signal and suppresses discrete noise interference from the source of weight assignment, providing core technical support for high-precision landslide identification.
- The proposed IHSA method has been verified by multi-dimensional validation, and its identification accuracy and reliability are significantly better than the traditional hot spot analysis method. In the preprocessing stage, multi-factor masking was applied to eliminate non-landslide-prone areas with slope < 10°, low-quality monitoring points with average coherence coefficient γ < 0.3, and river water body areas, which effectively reduces the scope of invalid calculation. After optimizing the extraction results with morphological operations and majority filtering, the method achieves a Precision of 90.20% (an increase of 53.61 percentage points compared with the traditional method) and a Recall of 92.00%. While ensuring the integrity of landslide identification, it greatly reduces the false positive rate.
- The IHSA method has excellent boundary extraction performance and effectively solves the problems of boundary burrs and internal voids in the traditional method. The extracted landslide boundaries have high consistency with the ground truth boundaries from manual interpretation, with smooth and continuous edges. It can completely retain the spatial morphological characteristics of landslides and accurately reflect the actual distribution range of landslides, further verifying the practicability and superiority of the method in automatic landslide extraction.
- The improved automatic landslide extraction method proposed in this study does not rely on artificial intelligence model training. For regions with different climatic backgrounds, topographic conditions and landslide development types, regional adaptation can be realized by adaptively adjusting the preprocessing screening and spatial weight configuration parameters according to local topographic threshold standards and the spatial distribution characteristics of time-series InSAR coherence.
- Although the IHSA method exhibits satisfactory identification, accuracy and application performance in the deeply incised Yangtze River valley, several limitations remain. Firstly, the method highly depends on the quality of time-series InSAR deformation inversion results, and poor data quality will restrict the stable operation of the model. Secondly, the area-descending priority rule adopted in morphological optimization is designed to preferentially guarantee the morphological integrity of medium and large landslides with high disaster prevention significance, which inevitably reduces the detection sensitivity to small-size landslides. Future research will introduce high-resolution SAR datasets (e.g., LuTan and ALOS-2, with a spatial resolution finer than 10 m) and establish multi-scale adaptive morphological rules, so as to compensate for the deficiency in the identification of micro and small landslides.

## Figures and Tables

**Table 1:** Key parameters and characteristics Sentinel-1A SAR sensors.

Satellite	Institution	Satellite Launch Time	Wave Band (Wavelength/cm)	Incidence Angle	Mode	Resolution	Period
Sentinel-1A	ESA	2014/04	C(5.6)	29–46	Interferometric Wide (IW)	2.32 × 13.97	12

**Table 2:** Basic information of the SAR data used herein.

Satellite	Orbit Direction	Path	Frame	Image Number	∆T ^1^ (Days)	$B_{⊥}$ ^2^ (m)	Master (YYYYMMDD)	Start Stop Time
Sentinel-1A	Ascending	157	92	244	12–36	0.3–250	20200101	20150409–20250122

Note: ^1^ represents temporal baseline, and ^2^ represents spatial baseline.

**Table 3:** Comparison of Landslide Identification Accuracy between the Improved IHSA Method and the Traditional Method.

Evaluation Metric	IHSA Method	Original Traditional Method
Total number of automatically extracted landslides	51	123
Number of correctly identified landslides (TP)	46	45
Number of falsely identified landslides (FP)	5	78
Number of missed landslides (FN)	4	5
Precision	90.20%	37.40%
Recall	92.00%	90.00%
F1-Score	91.09%	52.17%

## Data Availability

The Sentinel-1A SAR data used in this study are copyrighted by the European Space (https://dataspace.copernicus.eu, accessed on 1 March 2025). The GAMMA commercial software was obtained from https://www.gamma-rs.ch/gamma-software, accessed on 1 August 2023.

## References

[1] Friele P., Chiarle M. (2002). Landslide Risk Assessment and Management: An Overview. Eng. Geol..

[2] Hanssen R.F. (2001). Radar Interferometry: Data Interpretation and Error Analysis.

[3] Chen L., Zhao C., Chen H., Kang Y., Li B., Liu X. (2023). The Detection and Control Factor Analysis of Active Landslides in Guizhou Province, China, Using Sentinel-1 SAR Imagery. Remote Sens..

[4] Chen L., Zhao C., Kang Y., Chen H., Yang C., Li B., Liu Y., Xing A. (2020). Pre-Event Deformation and Failure Mechanism Analysis of the Pusa Landslide, China with Multi-Sensor SAR Imagery. Remote Sens..

[5] Ouchi K. (2013). Recent Trend and Advance of Synthetic Aperture Radar with Selected Topics. Remote Sens..

[6] Morishita Y., Lazecky M., Wright T.J., Weiss J.R., Elliott J.R., Hooper A. (2020). LiCSBAS: An Open-Source InSAR Time Series Analysis Package Integrated with the LiCSAR Automated Sentinel-1 InSAR Processor. Remote Sens..

[7] Liu X., Zhao C., Zhang Q., Lu Z., Li Z., Yang C., Zhu W., Liu-Zeng J., Chen L., Liu C. (2021). Integration of Sentinel-1 and ALOS/PALSAR-2 SAR datasets for mapping active landslides along the Jinsha River corridor, China. Eng. Geol..

[8] Zhang C., Li Z., Yu C., Chen B., Ding M., Zhu W., Yang J., Liu Z., Peng J. (2022). An integrated framework for wide-area active landslide detection with InSAR observations and SAR pixel offsets. Landslides.

[9] Song Y., Guo J., Wu G., Ma F., Li F. (2024). Automatic recognition of landslides based on YOLOv7 and attention mechanism. J. Mt. Sci..

[10] Lambert Z., Antoine R., Mauget C., Guilbert V., Beaucamp B., Aubin L., Davidson R., Costa S., Maquaire O., Fauchard C. (2026). A new automatic approach based on visible-thermal infrared data fusion to detect geological fracturation: The case of the Vaches Noires badland, Normandy, France. Landslides.

[11] Han J., Guo X., Jiao R., Nan Y., Yang H., Ni X., Zhao D., Wang S., Ma X., Yan C. (2023). An Automatic Method for Delimiting Deformation Area in InSAR Based on HNSW-DBSCAN Clustering Algorithm. Remote Sens..

[12] Zhang Y., Li Y., Meng X., Liu W., Wang A., Liang Y., Su X., Zeng R., Chen X. (2023). Automatic Mapping of Potential Landslides Using Satellite Multitemporal Interferometry. Remote Sens..

[13] Shen Y., Da K., Wu M., Zhou G., Wang M., Wang T., Xu Q. (2022). Rapid and automatic detection of new potential landslide based on phase-gradient DInSAR. IEEE Geosci. Remote Sens. Lett..

[14] Lu P., Bai S., Tofani V., Casagli N. (2019). Landslides detection through optimized hot spot analysis on persistent scatterers and distributed scatterers. ISPRS J. Photogramm. Remote Sens..

[15] Rosen P.A., Hensley S., Joughin I.R., Li F.K., Madsen S.N., Rodriguez E. (2002). Synthetic aperture radar interferometry. Proc. IEEE.

[16] Chowdhuri I., Pal S.C., Roy P. (2022). Mapping of earthquake hotspot and coldspot zones for identifying potential landslide hotspot areas in the himalayan region. Bull. Eng. Geol. Environ..

[17] Xiao S., Liu D., Hu Z. (2010). A Comparative Engineering Geological Study of Three Typical Reservoir-Type Bedding Rock Landslides in the World. J. Eng. Geol..

[18] Lu S. (2011). Study on Development Law and Instability Mechanism of Bedding Landslides Along the Three Gorges Reservoir Bank. Master’s Thesis.

[19] Xing L.X. (2012). Study on Genetic Mechanism and Prediction of Typical Accumulation Landslide in the Three Gorges Reservoir Area. Master’s Thesis.

[20] Xiang F., Zhu L., Wang C., Li Y., Yang W. (2005). Chronological Comparison Method of Terraces in the Three Gorges Area of the Yangtze River and Its Significance. J. Chengdu Univ. Technol. (Nat. Sci. Ed.).

[21] Chen S.F. (2022). Dynamic Characteristics and Trend Prediction of Jiuxianping Landslide in Yunyang, Three Gorges Reservoir Area. Master’s Thesis.

[22] Yang H., Tang M.G., Xu Q., Li W.L. (2021). Research of Statistical Characteristics of Deformation of Landslides in the Three Gorges Reservoir Area of the Yangtze River. J. Catastrophol..

[23] Deng M.L., Yi Q.L., Han B., Zhou J., Li Z.J., Zhang F.L. (2019). Analysis of surface deformation law of Muyubao landslide in the Three Gorges Reservoir Area of Yangtze River. Rock Soil Mech..

[24] Wang Q. (2018). Study on Regionalization Analysis of Landslide Susceptibility in the Three Gorges Reservoir Area of the Yangtze River. Master’s Thesis.

[25] Hua S. (2015). Study on Multi-stage Genetic Mechanism and Evolution Law of Huangtupo Landslide in the Three Gorges Reservoir Area. Doctoral Dissertation.

[26] Zhu D.P. (2010). Study on Reactivation Mechanism and Deformation Prediction of Typical Accumulation Landslide in the Three Gorges Reservoir Area. Doctoral Dissertation.

[27] Lu Z., Dzurisin D., Jung H., Zhang J., Zhang Y. (2010). Radar image and data fusion for natural hazards characterization. Int. J. Image Data Fusion.

[28] Funning G., Garcia A. (2019). A systematic study of earthquake detectability using Sentinel−1 interferometric wide-swath data. Geophys. J. Int..

[29] Torres R., Snoeij P., Geudtner D. (2012). GMES Sentinel-1A mission. Remote Sens. Environ..

[30] Berardino P., Fornaro G., Lanari R., Sansosti E. (2002). A new algorithm for surface deformation monitoring based on small baseline differential SAR interferograms. IEEE Trans. Geosci. Remote Sens..

[31] Ferretti A., Prati C., Rocca F. (2001). Permanent scatterers in SAR interferometry. IEEE Trans. Geosci. Remote Sens..

[32] Fuhrmann T., Garthwaite M.C. (2019). Resolving Three-Dimensional Surface Motion with InSAR: Constraints from Multi-Geometry Data Fusion. Remote Sens..

[33] Goldstein R.M., Werner C.L. (1998). Radar interferogram filtering for geophysical applications. Geophys. Res. Lett..

[34] Werner C., Wegmuller U., Strozzi T., Wiesmann A. Interferometric Point Target Analysis for Deformation Mapping. Proceedings of the 2003 IEEE International Geoscience and Remote Sensing Symposium.

[35] Doin M.P., Lasserre C., Peltzer G., Cavalié O., Doubre C. (2009). Corrections of stratified tropospheric delays in SAR interferometry: Validation with global atmospheric models. J. Appl. Geophys..

[36] Pepe A., Lanari R. (2006). On the extension of the minimum cost flow algorithm for phase unwrapping of multi-temporal differential SAR interferograms. IEEE Trans. Geosci. Remote Sens..

[37] Liu P., Li Z., Hoey T., Kincal C., Zhang J., Zeng Q., Muller J.P. (2013). Using advanced InSAR time series techniques to monitor landslide movements in Badong of the three gorges region, China. Int. J. Appl. Earth Obs. Geoinf..

[38] Hooper A., Segall P., Zebker H. (2007). Persistent scatterer InSAR for crustal deformation analysis, with application to Volcán Alcedo, Galápagos. J. Geophys. Res. Solid Earth.

[39] Bekaert D.P., Handwerger A.L., Agram P., Kirschbaum D.B. (2020). InSAR-based detection method for mapping and monitoring slow-moving landslides in remote regions with steep and mountainous terrain: An application to Nepal. Remote Sens. Environ..

[40] Xun Z., Zhao C., Kang Y., Liu X., Liu Y., Du C. (2022). Automatic Extraction of Potential Landslides by Integrating an Optical Remote Sensing Image with an InSAR-Derived Deformation Map. Remote Sens..

[41] Li Z., Dai K., Deng J., Liu C., Shi X., Tang G., Yin T. (2023). Identifying Potential Landslides in Steep Mountainous Areas Based on Improved Seasonal Interferometry Stacking-InSAR. Remote Sens..

[42] Getis A., Ord J.K. (1992). The Analysis of Spatial Association by Use of Distance Statistics. Geogr. Anal..

[43] Getis A., Ord J.K., Longley P.A., Batty M. (1996). Local spatial statistics: An overview. Spatial Analysis: Modelling in a GIS Environment.

[44] Serra J. (1982). Image Analysis and Mathematical Morphology.

[45] Wells D.C. The Mode Filter: A Nonlinear Image Processing Operator. Proceedings of the SPIE Instrumentation in Astronomy III.

[46] Sahrane R., Bounab A., El Kharim Y., Obda O., El Miloudi Y., Mihraje A.I., Afi M.E. (2025). Landslide–Anthropogenic Interactions in Urban Areas: A Multidisciplinary Case Study from Taounate, Morocco. Geotech. Geol. Eng..

[47] Dong J., Zhang L., Liao M., Gong J. (2019). Improved correction of seasonal tropospheric delay in InSAR observations for landslide deformation monitoring. Remote Sens. Environ..

